# Efficacy and Safety of a Continuous Intravenous Insulin Protocol Modified for East Asians in Postoperative Glycemic Management Following Pancreatectomy

**DOI:** 10.7759/cureus.84527

**Published:** 2025-05-21

**Authors:** Hironobu Sasaki, Kazuma Yagi, Ryota Kogure, Masayuki Honda, Dal Ho Kim

**Affiliations:** 1 Division of Diabetes, Metabolism and Endocrinology, Department of Internal Medicine, Sainokuni Higashiomiya Medical Center, Saitama, JPN; 2 Department of Surgery, Sainokuni Higashiomiya Medical Center, Saitama, JPN

**Keywords:** diabetes, glycemic control, insulin protocol, intravenous insulin infusion, pancreatectomy, postoperative hyperglycemia

## Abstract

Introduction

Glycemic control following pancreatectomy presents challenges, especially in patients with diabetes due to a lack of endogenous insulin, however, optimal management remains unclear. This study evaluated the efficacy and safety of a continuous intravenous insulin infusion protocol employed at our institution, in comparison with conventional glycemic control in patients with pancreatectomy.

Materials and methods

Sixty-one patients with preoperative glycosylated hemoglobin (HbA1c) of 6.5% or higher, on diabetes medications, or who underwent total pancreatectomy were included. Patients were categorized into three groups: insulin protocol (IP group, *n *= 24), subcutaneous injection (SI group, *n *= 15), and continuous intravenous insulin infusion based on the empirical control (EC group, *n *= 22). The primary outcomes were average blood glucose levels and the proportion of achievement within the target blood glucose range (140-180 mg/dl). Additionally, factors associated with the insulin dose in the IP group were analyzed.

Results

At predefined time points, the IP group achieved a significantly higher proportion of the target blood glucose range than the SI group (46.2% vs. 31.6%, *p* = 0.01), with no significant difference in average blood glucose levels (164.1 ± 41.8 vs. 169.1 ± 51.0 mg/dl, *p *= 0.50). During the 60-hour period following the initiation of frequent blood glucose measurements, the IP group demonstrated significantly reduced average blood glucose levels than the EC group (170.1 ± 56.0 vs. 175.5 ± 43.5 mg/dl, *p* <0.001), despite significantly longer measurement intervals (1.5 ± 0.7 vs. 1.2 ± 0.7 hours, *p* <0.001). However, there was no significant difference in the proportion of the target blood glucose range between the IP and EC groups (37.2% vs. 41.0%, *p* = 0.11). Aspartate transaminase and alanine transferase levels on postoperative day one were positively correlated with the average insulin dose in the IP group (both *R* = 0.45, *p* = 0.03).

Conclusions

This IP helped stabilize blood glucose levels compared to subcutaneous injections and improved glycemic control more effectively than empirically administered continuous intravenous insulin infusion. Postoperative elevations in liver enzymes may serve as predictors of increased insulin requirements.

## Introduction

The postoperative period is prone to elevated blood glucose levels due to sympathetic activation caused by surgical stress and an increase in insulin antagonist hormones such as catecholamines, growth hormone, and glucocorticoids [1]. Additionally, inflammatory cytokines, medications affecting the sympathetic nervous system, and active nutritional supplementation further complicate glycemic control [2]. Hyperglycemia exacerbates inflammation and oxidative stress, creating a vicious cycle that further promotes elevated blood glucose levels [3]. Postoperative hyperglycemia has been linked to adverse outcomes, including impaired wound healing, increased infection rates, and higher mortality [4,5]. Particularly in patients undergoing pancreatectomy, the partial or complete loss of insulin secretion following surgery can easily lead to glucose disturbance [6], necessitating meticulous glycemic management. Many of these patients also present with preoperative diabetes or impaired glucose tolerance [7], making postoperative glycemic control even more challenging. Postoperative blood glucose management is performed using intensive insulin therapy (IIT) or sliding scale subcutaneous injections (SIs), as well as continuous intravenous insulin infusion [8]. Although there are some reports that an artificial pancreas can effectively maintain blood glucose levels within the normal range after pancreatectomy [2,9], a standardized, simple, and broadly applicable method for postoperative glycemic control has yet to be established.

The Leuven I trial showed that IIT, a strict glycemic control strategy, was effective in reducing mortality in critically ill patients [10], however, severe hypoglycemia was increased by IIT, including in the VISEP (Volume Substitution and Insulin Therapy in Severe Sepsis) [11] and Glucontrol studies [12]. The NICE-SUGAR (Normoglycemia in Intensive Care Evaluation-Survival Using Glucose Algorithm Regulation) trial also found higher 90-day mortality and increased risk of severe hypoglycemia in critically ill patients in the IIT group with a target level of 81-108 mg/dl compared to the conventional control group with a target level of 144-180 mg/dl [13]. Subsequent meta-analyses have confirmed that IIT is not effective in critically ill patients and increases the risk of severe hypoglycemia [14-16]. Accordingly, the American Diabetes Association (ADA) now recommends a target glucose range of 140-180 mg/dl for critically ill patients admitted to the intensive care unit (ICU) [17].

A well-established protocol for continuous intravenous insulin infusion used in the ICU setting was developed by Yale University [18]. The Yale protocol, originally developed for Westerners, was modified at Keio University to better accommodate East Asians, who are generally less obese and more insulin-sensitive, and was further innovated to adjust the target blood glucose range from 100-139 mg/dl to 140-180 mg/dl in accordance with the current ADA guideline [19]. This simplified protocol, which adjusts insulin based on both current blood glucose levels and their rate of change, was verified to be as effective and safe as conventional methods of glycemic control, which involve continuous insulin infusion adjusted according to physicians’ clinical judgment while reducing the burden on medical staff for insulin adjustment when adapted for patients undergoing open-heart surgery [19]. However, the efficacy and safety of this protocol in other surgical procedures have not been fully elucidated. We hypothesized that applying this protocol to East Asian patients would ensure similar efficacy and safety in glycemic control, regardless of the underlying disease, compared to non-protocol-based management. To explore this hypothesis, we applied the protocol to patients undergoing pancreatectomy.

## Materials and methods

Patients’ selection

This retrospective study examined the efficacy and safety of the continuous intravenous insulin infusion protocol currently employed at our institution, in comparison with conventional methods of glycemic control in patients undergoing pancreatectomy.

**Figure 1:**
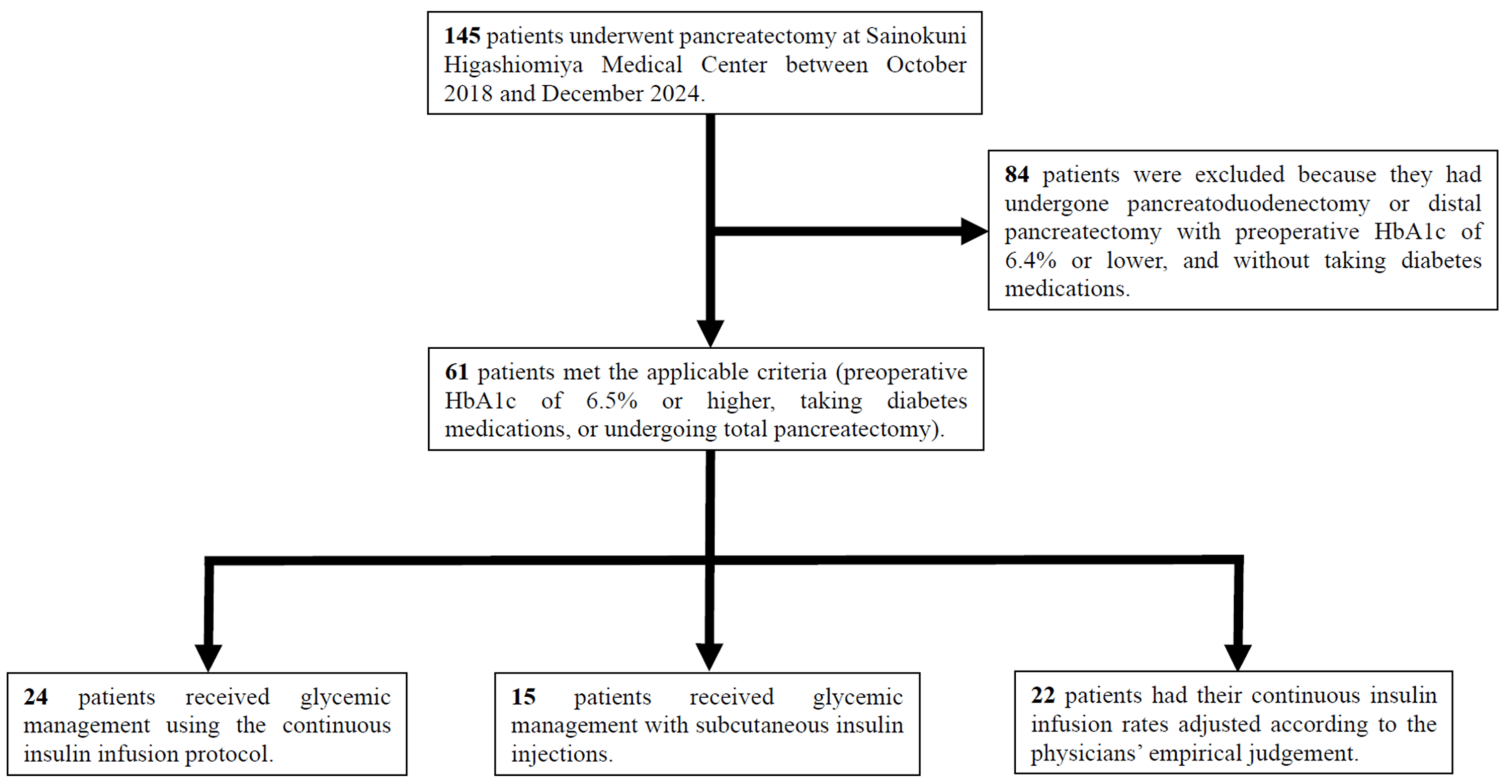
Flowchart of study participants. HbA1c, glycosylated hemoglobin.

From October 2018 to December 2025, 145 patients underwent pancreatectomy at Sainokuni Higashiomiya Medical Center, Saitama, Japan. Of these, 61 patients met the inclusion criteria: 1) preoperative glycosylated hemoglobin (HbA1c) of 6.5% or higher, 2) taking diabetes medications, or 3) undergoing total pancreatectomy. Among patients who underwent pancreatoduodenectomy or distal pancreatectomy, 84 were excluded due to preoperative HbA1c <6.5% and no use of diabetes medications. All patients were admitted to the ICU postoperatively and received blood glucose management by one of three methods: 1) continuous intravenous insulin infusion using insulin protocol (IP): IP group, 2) SI with sliding scale insulin or IIT: SI group, or 3) continuous intravenous insulin infusion based on the physicians' empirical control (EC): EC group. The flowchart of study participants is shown in Figure 1.

In the IP group, continuous intravenous insulin infusion was started at the time of the ICU admission after surgery, and the infusion rate and blood glucose measurement interval every one to three hours were determined. In the SI group, blood glucose was measured four times daily (before breakfast, lunch, dinner, and bedtime) starting from ICU admission, and insulin was administered subcutaneously based on blood glucose levels. In the EC group, frequent blood glucose measurements were started every hour after admission to the ICU following surgery. Continuous intravenous insulin infusion was started at a rate of 1 unit/hour once blood glucose levels exceeded 180 mg/dl. Thereafter, measurement intervals were allowed to extend up to every three hours, and the insulin dose was adjusted to the timing of blood glucose measurements. Patients’ characteristics are presented in Table 1.

**Table 1:** Patients’ characteristics. Values are mean ± standard deviation or *n* (%). ^a^ Timing of blood sampling (i.e., fasting or postprandial) was not determined. ^b^ Inflammatory pseudotumor, diffuse large B-cell lymphoma, descending colon cancer with pancreatic invasion, gallbladder cancer, gastric cancer with pancreatic invasion, xanthogranulomatous pancreatitis, intraductal tubular adenoma, *n*=1 for each. ^c^ Chi-square test, ^d^ Fisher's exact test, ^e^ Chi-square value, ^f^ H value, ^g^ Mann-Whitney U value. BMI, Body mass index; EC, Empirical control; HbA1c, Glycosylated hemoglobin; eGFR, Estimated glomerular filtration rate; IP, Insulin protocol; SI, Subcutaneous injection.

	IP group	SI group	EC group	P value	Test statistic
N (male/female)	24 (12/12)	15 (10/5)	22 (13/9)	0.58^ c^	1.09^ e^
Age, years	74.5 ± 7.7	70.8 ± 10.5	74.0 ± 8.3	0.47	1.51^ f^
BMI, kg/m^2^	22.2 ± 3.4	21.8 ± 2.6	22.7 ± 3.3	0.57	1.12^ f^
Blood glucose level, mg/dl ^a^	165.3 ± 60.3	136.5 ± 40.3	153.0 ± 46.5	0.28	2.57^ f^
HbA1c, %	7.2 ± 0.9	6.9 ± 1.0	7.0 ± 1.1	0.32	2.29^ f^
eGFR, ml/min/1.73m^2^	77.6 ± 26.0	70.2 ± 24.3	71.1 ± 20.5	0.44	1.63^ f^
History of diabetes, n (%)	22 (91.7)	14 (93.3)	21 (95.5)	1^d^	-
Duration of frequent blood glucose measurement, hours	72.6 ± 68.6	-	56.2 ± 16.9	0.47	231^ g^
Preoperative diabetes therapy, n (%)					
Insulin	7 (29.2)	3 (20.0)	5 (22.7)	0.79^ c^	0.48^ e^
Oral hypoglycemic agent	13 (54.2)	7 (46.7)	10 (45.5)	0.82^ c^	0.40^ e^
Clinical diagnosis, n (%)					
Pancreatic cancer	13 (54.2)	6 (40.0)	9 (40.9)	0.58^ c^	1.09^ e^
Bile duct cancer	5 (20.8)	1 (6.7)	4 (18.2)	0.61^ d^	-
Duodenal papillary cancer	3 (12.5)	1 (6.7)	2 (9.1)	1^ d^	-
Intraductal papillary mucinous neoplasm	3 (12.5)	0 (0)	3 (13.6)	0.42^ d^	-
Non-functional neuroendocrine tumor	0 (0)	2 (13.3)	2 (9.1)	0.17^ d^	-
Other ^b^	0 (0)	5 (33.3)	2 (9.1)	0.0033^ d^	-
Operative procedure, n (%)					
Pancreatoduodenectomy	13 (54.2)	9 (60.0)	16 (72.7)	0.42^ c^	1.73^ e^
Distal pancreatectomy	6 (25.0)	5 (33.3)	4 (18.2)	0.57^ c^	1.11^ e^
Total pancreatectomy	5 (20.8)	1 (6.7)	2 (9.1)	0.43^ d^	-
Complication, n (%)					
Hypertension	18 (75.0)	11 (73.3)	18 (81.8)	0.80^ c^	0.46^ e^
Dyslipidemia	12 (50.0)	10 (66.7)	14 (63.6)	0.51^ c^	1.36^ e^
Cerebrovascular disease	3 (12.5)	1 (6.7)	2 (9.1)	1^ d^	-
Coronary artery disease	1 (4.2)	2 (13.3)	3 (13.6)	0.55^ d^	-
Preoperative cholangitis	3 (12.5)	5 (33.3)	5 (22.7)	0.32^ d^	-

Insulin infusion 

In the IP group, the change in blood glucose levels per hour was calculated for each blood glucose measurement after the initiation of continuous intravenous insulin infusion, and the corresponding cell in Table 2 was identified based on the current blood glucose levels.

**Table 2:** Change in blood glucose levels per hour (X) from previous blood glucose levels. Δ, Changes in insulin infusion rate. Source: Inaishi et al. [19].

Current blood glucose level (mg/dl)	X≦-100	-100＜X≦-75	-75＜X≦-50	-50＜X≦-25	-25＜X≦0	0＜X≦25	25＜X≦50	50＜X
80-139	-2Δ	-2Δ	-2Δ	-2Δ	-Δ	0	0	0
140-179	-2Δ	-2Δ	-2Δ	-Δ	-Δ	0	Δ	Δ
180-199	-2Δ	-2Δ	-Δ	-Δ	0	Δ	Δ	2Δ
200-	-2Δ	-Δ	0	0	Δ	2Δ	2Δ	2Δ

Then, the amount of insulin adjustment required to correct the current insulin infusion rate was confirmed based on Table 3, and a new insulin infusion rate was determined. Even when the calculated infusion rate fell below 0 units/hour, the continuous intravenous insulin infusion was maintained at a minimum dose of 0.1 units/hour. When blood glucose levels dropped below 80 mg/dl, continuous intravenous insulin infusion was stopped, 40 ml of 50% glucose was administered intravenously, and blood glucose levels were rechecked 15 minutes later. If blood glucose levels were still less than 80 mg/dl, an intravenous glucose infusion was repeated, followed by blood glucose measurement 15 minutes later. If blood glucose levels were 80 mg/dl or higher, blood glucose levels were measured again 30 minutes later, and when they still exceeded 80 mg/dl, continuous intravenous insulin infusion was reinitiated. The restarted insulin infusion rate was set to the rate before discontinuation with a change of -2⊿ in Table 3.

**Table 3:** Change in insulin infusion rate from the current rate. Δ, Changes in insulin infusion rate. Source: Inaishi et al. [19].

Current insulin infusion rate (units/hour)	Δ (units/hour)	2Δ (units/hour)
<1.0	0.3	0.6
1.1-2.0	0.4	0.8
2.1-3.0	0.6	1.2
3.1-6.0	1.0	2.0
6.1-10.0	1.5	3.0
10.1-15.0	2.0	4.0
>15.1	3.0	6.0

Outcome measure

Patients’ data including age, gender, diagnosis, surgical procedure, complications, blood data, and the insulin dose, were extracted from medical records. Blood glucose levels were measured using either an arterial blood gas analysis (ABL800 FLEX; Radiometer, Copenhagen, Denmark) or point-of-care testing (STAT STRIP Xpress; Nova Biomedical, Waltham, MA, USA). Blood glucose levels at nine points from before breakfast on postoperative day (POD) one to before breakfast on POD three (i.e., POD one before breakfast, POD one before lunch, POD one before dinner … POD two bedtime, POD three before breakfast) were compared between the IP and SI groups. The average blood glucose levels for each group were calculated. Setting 140-180 mg/dl as the target blood glucose range, the proportions of blood glucose levels above, within, and below the target range and hypoglycemia (<70 mg/dl) were determined. Likewise, the IP and EC groups were compared in terms of blood glucose levels and the insulin dose up to 60 hours after the initiation of frequent blood glucose measurements. In addition, factors contributing to the insulin dose under the IP were investigated based on postoperative blood data.

Statistical analysis

Continuous variables were expressed as mean ± standard deviation and categorical variables as percentages (%). Differences in continuous variables were analyzed using the Kruskal-Wallis test for comparisons among three groups and the Mann-Whitney U test for comparisons between two groups. Categorical variables were compared using either the Chi-square test or Fisher’s exact test. Simple linear regression analyses were conducted to identify factors contributing to the insulin dose in the IP. All analyses were performed using IBM SPSS Statistics for Windows, Version 30, (IBM Corp., Armonk, NY, USA) and a *p* value <0.05 was considered statistically significant.

Ethical considerations

This study was approved by the Ethics Committee of Sainokuni Higashiomiya Medical Center (Approval Date: March 12, 2025, Approval No. 66) and was conducted in accordance with the principles of the Declaration of Helsinki.

## Results

Patients’ characteristics

The characteristics of the study participants are summarized in Table 1. Most patients had been diagnosed with diabetes prior to surgery, except for a few who underwent total pancreatectomy. In the IP group, frequent blood glucose measurements were completed within 24 hours in three patients and within 48 hours in 11 patients. In the EC group, this occurred within 24 hours in two patients and within 48 hours in three patients. Pancreatic cancer was the most common diagnosis across all groups, and pancreatoduodenectomy was the most frequently performed surgical procedure. More than 70% of patients in all groups had hypertension, which was the most common complication. No significant differences were observed among the groups for any parameters, except for the proportion of patients with "other" diseases in the clinical diagnosis category.

Comparison between the IP and SI groups

Blood glucose levels measured 158 times in the IP group and 133 times in the SI group were compared. There was no difference in average blood glucose levels between the two groups (164.1 ± 41.8 vs. 169.1 ± 51.0 mg/dl, *p* = 0.50, Table 4). However, the IP group showed a significant improvement in the proportion of blood glucose levels within the target range compared to the SI group (46.2% vs. 31.6%, *p* = 0.01). The proportions of values above (26.6% vs. 36.1%, *p* = 0.08) and below (27.2% vs. 32.3%, *p* = 0.34) the target range did not differ significantly between the two groups. No hypoglycemic events (<70 mg/dl) were observed in either group at the time of the observation.

**Table 4:** Comparison of glycemic control between the IP and SI groups at nine time points up to the morning of postoperative day three. Values are mean ± standard deviation or *n* (%). ^a^ Chi-square test, ^b^ Fisher's exact test, ^c^ Mann-Whitney U value, ^d^ Chi-square value. IP, Insulin protocol; SI, Subcutaneous injection.

	IP group	SI group	*P* value	Test statistic
Average blood glucose level, mg/dl	164.1 ± 41.8	169.1 ± 51.0	0.50	10020.5^ c^
Above the target blood glucose range (181 mg/dl or higher), times (%)	42 (26.6)	48 (36.1)	0.08^ a^	3.06^ d^
Within the target blood glucose range (140-180 mg/dl), times (%)	73 (46.2)	42 (31.6)	0.01^ a^	6.46^ d^
Below the target blood glucose range (139 mg/dl or less), times (%)	43 (27.2)	43 (32.3)	0.34^ a^	0.91^ d^
Hypoglycemia (less than 70 mg/dl), times (%)	0 (0)	0 (0)	1 ^b^	-

Comparison between the IP and EC groups 

From the initiation of frequent blood glucose measurements to 60 hours postoperatively, a total of 787 and 986 blood glucose measurements were obtained in the IP and EC groups, respectively. The IP group showed significantly lower average blood glucose levels than the EC group (170.1 ± 56.0 vs. 175.5 ± 43.5 mg/dl, *p* <0.001, Table 5). However, the average blood glucose measurement interval was significantly longer in the IP group (1.5 ± 0.7 vs. 1.2 ± 0.7 hours, *p* <0.001). The average insulin infusion rate was significantly lower in the IP group than in the EC group (0.8 ± 0.8 vs. 1.4 ± 1.3 units/hour, *p* <0.001), although the maximum infusion rate did not significantly differ between the two groups (2.3 ± 1.1 vs. 3.2 ± 1.6 units/hour, *p* = 0.06).

**Table 5:** Comparison between the IP and EC groups over 60 hours following the initiation of frequent blood glucose measurements. Values are mean ± standard deviation or *n* (%). ^a^ Chi-square test, ^b^ Fisher's exact test, ^c^ Mann-Whitney U value, ^d^ Chi-square value. EC, Empirical control; IP, Insulin protocol.

	IP group	EC group	*P* value	Test statistic
Average blood glucose level, mg/dl	170.1 ± 56.0	175.5 ± 43.5	<0.001	350122.5^ c^
Average glucose measurement interval, hours	1.5 ± 0.7	1.2 ± 0.7	<0.001	282438.5^ c^
Average insulin infusion rate, units/hour	0.8 ± 0.8	1.4 ± 1.3	<0.001	548224.5^ c^
Maximum insulin infusion rate, units/hour	2.3 ± 1.1	3.2 ± 1.6	0.06	177.5^ c^
Above the target blood glucose range (181 mg/dl or higher), times (%)	273 (34.7)	386 (39.1)	0.054^ a^	3.73^ d^
Within the target blood glucose range (140-180 mg/dl), times (%)	293 (37.2)	404 (41.0)	0.11^ a^	2.57^ d^
Below the target blood glucose range (139 mg/dl or less), times (%)	221 (28.1)	196 (19.9)	<0.001^a^	16.37^ d^
Hypoglycemia (less than 70 mg/dl), times (%)	5 (0.6)	2 (0.2)	0.14 ^b^	-

The proportions of blood glucose above (34.7% vs. 39.1%, *p* = 0.054) and within (37.2% vs. 41.0%, *p* = 0.11) the target range were not significantly different between the two groups. The proportions of values below the target range were significantly higher in the IP group than in the EC group (28.1% vs. 19.9%, *p* <0.001), but the incidence of hypoglycemia (<70 mg/dl) was not significantly different between the two groups (0.6% vs. 0.2%, *p* = 0.14).

**Figure 2:**
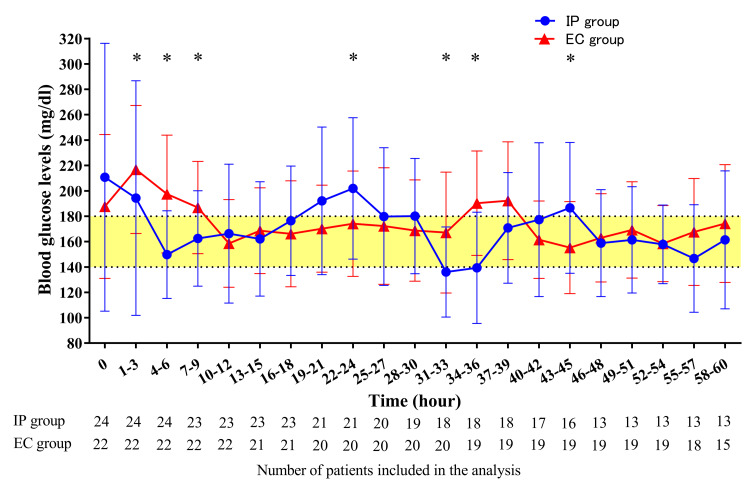
Trend in postoperative blood glucose levels over 60 hours following the initiation of frequent measurements. Values are mean ± standard deviation. * *p* <0.05 The number of patients included in the analysis is indicated below the figure. EC, Empirical control; IP, Insulin protocol.

The three-hourly average blood glucose comparison revealed that the IP group showed significantly lower average blood glucose levels than the EC group from one to nine hours after the initiation of frequent blood glucose measurements, reaching the target range immediately (Figure 2).

Factors affecting the insulin dose when using the IP 

To identify factors affecting the insulin dose in the IP group, a simple linear regression analysis was performed using POD one blood data in relation to both average and maximum insulin infusion rates. As a result, aspartate transaminase (AST) and alanine transferase (ALT) levels on POD one were positively correlated with the average insulin infusion rate (both *R* = 0.45, *p* = 0.03, Table 6). Likewise, AST and ALT levels on POD one were positively correlated with the maximum insulin infusion rate (both *R* = 0.51, *p* = 0.01).

**Table 6:** Simple linear regression analysis between postoperative day one blood data and both the average and maximum insulin infusion rates in the IP group. Alb, Albumin; ALP, Alkaline phosphatase; ALT, Alanine transferase; AST, Aspartate transaminase; B.AMY, Blood amylase; CRP, C-reactive protein; D.Bil, Direct bilirubin; eGFR, Estimated glomerular filtration rate; ɤ-GTP, Gamma-glutamyl transferase; Hb, Hemoglobin; IP, Insulin protocol; K, Potassium; Na, Natrium; P.AMY, Pancreatic amylase; T.Bil, Total bilirubin; TP, Total protein; WBC, White blood cell.

Variables	Average insulin infusion rate		Maximum insulin infusion rate	
	R	*P* value	R	*P* value
WBC, /mm^3^	0.13	0.55	0.01	0.96
Hb, g/dl	-0.06	0.77	-0.12	0.57
TP, g/dl	0.08	0.70	-0.01	0.96
Alb, g/dl	0.10	0.66	0.08	0.72
T.Bil, mg/dl	0.32	0.13	0.33	0.11
D.Bil, mg/dl	0.28	0.18	0.27	0.20
AST, U/l	0.45	0.03	0.51	0.01
ALT, U/l	0.45	0.03	0.51	0.01
ALP, U/l	0.01	0.97	0.05	0.82
ɤ-GTP, U/l	0.01	0.97	0.02	0.91
B.AMY, U/l	0.25	0.23	0.29	0.16
P.AMY, U/l	-0.03	0.89	-0.13	0.56
eGFR, ml/min/1.73m^2^	0.23	0.27	0.14	0.51
Na, mEq/l	0.13	0.53	0.25	0.24
K, mEq/l	0.21	0.32	0.23	0.29
CRP, mg/dl	-0.34	0.11	-0.25	0.23
Lactate, mmol/l	0.04	0.84	0.09	0.67

## Discussion

In this study, we found that the IP group had a significantly higher proportion of blood glucose levels within the target range compared to the SI group, despite no significant difference in average blood glucose levels between the two groups. That is, the implementation of this continuous intravenous IP in patients with pancreatectomy may reduce blood glucose fluctuations compared to SI. To the best of our knowledge, there is no direct evidence that postoperative continuous intravenous insulin administration reduces glycemic variability compared to SI. On the contrary, several studies have reported that although continuous intravenous insulin infusion improves blood glucose levels more rapidly in critically ill patients [20], it is also associated with an increased risk of hypoglycemia and greater glycemic variability [20,21]. However, these findings were all limited to non-surgical cases, and it is considered difficult to directly apply them to post-surgical management, especially following pancreatectomy. Continuous intravenous insulin, if administered according to a well-designed protocol in patients with pancreatectomy, could contribute to achieving the target blood glucose levels and reducing glycemic variability through accurate dose adjustment. In addition, this protocol did not increase the incidence of blood glucose levels above the target range or hypoglycemia compared to SI, supporting its safety in postoperative care following pancreatectomy.

Another important finding was that the IP group exhibited significantly improved average blood glucose levels compared to the EC group, despite significantly longer blood glucose measurement intervals, although there was no significant difference in the proportion of blood glucose levels above or within the target range. Conventionally, the amount of intravenous insulin was adjusted based on the physician's orders, often relying on verbal instructions, which carry the risk of medical errors. This IP may help reduce such medical errors, as insulin adjustments can be made systematically without needing a direct physician’s order after initial dose determination. Notably, the fact that the IP group improved average blood glucose levels despite significantly longer intervals between measurements suggests effective glycemic management while reducing the burden on both medical staff and patients in terms of glucose measurement and insulin dose adjustments. From one to nine hours after the initiation of frequent blood glucose measurements, average blood glucose levels evaluated every three hours were significantly lower in the IP group than in the EC group. Moreover, average blood glucose levels rapidly reached the target range following the initiation of frequent blood glucose measurements in the IP group. In the EC group, continuous insulin infusion was initiated only after blood glucose exceeded 180 mg/dl, potentially resulting in delayed intervention. The ADA guideline states that insulin therapy should be initiated or intensified in critically ill patients when blood glucose levels exceed 180 mg/dl [17]. However, patients with post-pancreatectomy are particularly vulnerable to dysglycemia due to decreased insulin secretion [6]. Even when glucose levels are within the target range, starting low-dose insulin infusion upon ICU admission may be crucial for early glycemic stabilization, as implemented in the IP group.

Interestingly, we also found a positive correlation between liver enzyme levels (AST and ALT) on the day after surgery and insulin requirement when using this protocol. Insulin resistance is known to contribute to hepatic fat accumulation, inflammation, and fibrosis, leading to impaired liver function [22,23]. Elevated liver enzymes may serve as indicators of insulin resistance induced by surgical stress. Therefore, early postoperative monitoring of liver function could aid in predicting insulin needs and identifying patients at risk of poor glycemic control after pancreatectomy. When using this protocol, the presence of severe hepatic dysfunction on the day after surgery may lead to increased insulin requirements, necessitating careful monitoring for potential complications such as hypokalemia and fat accumulation.

This study was subject to certain limitations. First, the study's relatively small sample size may have included the effect of β-error, and assignment to each treatment group was based on physician judgment rather than randomization, possibly introducing selection bias. Second, we could not assess whether improvements in blood glucose metrics were associated with clinical outcomes such as infection rates, wound healing, length of hospital stay, or mortality. Third, potential confounders such as postoperative infections and nutritional management were not controlled for, and the use of multiple devices for blood glucose measurement may have introduced measurement errors that could have affected our results. Finally, the duration of frequent glucose measurements and intravenous insulin infusion varied among patients, and many cases were terminated before the end of the observation period, possibly affecting the assessment of the protocol's efficacy. To overcome these limitations, it is necessary to standardize the blood glucose measurement devices, establish clear criteria and observation schedules to prevent premature discontinuation of the intervention and conduct a larger-scale randomized prospective trial to evaluate the protocol’s impact on clinical outcomes.

## Conclusions

The application of the IP in patients with pancreatectomy improved the achievement of the target glycemic range compared to SI with an insulin sliding scale or IIT. Compared to empirically managed continuous intravenous insulin infusion, the IP resulted in better average blood glucose levels while reducing the burden of blood glucose measurements and insulin adjustment, without increasing the risk of hypoglycemia. Furthermore, elevated liver enzymes on POD one may be a predictor of increased insulin requirements, indicating the need to monitor potassium levels due to insulin’s effects.

These findings support that the IP is an effective and safe treatment strategy for postoperative glycemic management in East Asian patients undergoing pancreatectomy, as well as those previously evaluated after open-heart surgery.
